# Harnessing Liquiritigenin: A Flavonoid-Based Approach for the Prevention and Treatment of Cancer

**DOI:** 10.3390/cancers17142328

**Published:** 2025-07-13

**Authors:** Anjana Sajeev, Babu Santha Aswani, Mohammed S. Alqahtani, Mohamed Abbas, Gautam Sethi, Ajaikumar B. Kunnumakkara

**Affiliations:** 1 Cancer Biology Laboratory, Department of Biosciences and Bioengineering, Indian Institute of Technology Guwahati (IITG), Guwahati 781039, Assam, India; s.anjana@iitg.ac.in (A.S.); s.aswani@iitg.ac.in (B.S.A.); 2 Radiological Sciences Department, College of Applied Medical Sciences, King Khalid University, Abha 61421, Saudi Arabia; mosalqhtani@kku.edu.sa; 3 BioImaging Unit, Space Research Centre, Michael Atiyah Building, University of Leicester, Leicester LE1 7RH, UK; 4 Electrical Engineering Department, College of Engineering, King Khalid University, Abha 61421, Saudi Arabia; mabas@kku.edu.sa; 5 Department of Pharmacology, Yong Loo Lin School of Medicine, National University of Singapore, Singapore 117600, Singapore

**Keywords:** liquiritigenin, phytochemical, cancer therapy, signaling pathways, pharmacokinetics, chemosensitization

## Abstract

Background/Objectives: The integration of natural compounds in cancer research marked a crucial shift in the modern medical landscape, through a growing acknowledgment of their potential as efficient, less toxic, and cost-effective alternatives to contemporary chemotherapeutics. Liquiritigenin (LIQ) is a compound obtained from different plants, the most important being the *Glycyrrhiza* species, commonly known as licorice. Methods: This review compiles findings from previously published preclinical studies and experimental research articles focusing on LIQ’s pharmacological effects, with particular attention to its anticancer potential. The relevant literature was identified using established scientific databases and selected based on relevance to cancer biology and LIQ-associated signaling pathways. Results: LIQ demonstrates anti-oxidant, anti-inflammatory, and anti-proliferative effects. It exerts its potential anticancer activities by inducing apoptosis, preventing cell proliferation, and modulating various signaling pathways such as nuclear factor kappa-light-chain-enhancer of activated B cells (NF-κB), phosphoinositide 3 kinase (PI3K)/Akt/mammalian target of rapamycin (mTOR), mitogen-activated protein kinase (MAPK), and so on. Conclusions: LIQ represents a promising natural agent for cancer therapy, with evidence supporting its multifunctional role in targeting tumor growth and survival mechanisms. By providing a detailed analysis of LIQ, this review aims to highlight its therapeutic efficacy across various cancer types and emphasize its importance as a promising compound in cancer research. In addition, this review seeks to bridge the gap between traditional medicine and modern pharmacology and paves the way for LIQ’s clinical application in cancer therapy.

## Simple Summary

This review highlights the therapeutic potential of liquiritigenin (LIQ), a natural flavonoid primarily derived from licorice (*Glycyrrhiza* species), in cancer treatment. LIQ exhibits multiple biological activities, including anti-oxidant, anti-inflammatory, and anti-proliferative effects, contributing to its anticancer properties. This review discusses its ability to induce apoptosis, inhibit cell proliferation, and modulate key signaling pathways such as NF-κB, PI3K/Akt/mTOR, and MAPK. By consolidating current findings, this review highlights LIQ’s value as a promising, less toxic, and cost-effective anticancer agent, bridging traditional herbal medicine with modern pharmacology and supporting its future clinical application.

## 1. Introduction

Cancer remains a leading cause of morbidity and mortality, with the International Agency for Research and Cancer (IARC) reporting nearly 20 million new cases and 10 million deaths in 2022 [1]. Despite significant strides in conventional treatments, including surgery, chemotherapy, radiation therapy, and targeted therapies, these approaches are double-edged swords, with their efficacy being overshadowed by limited success in combating metastatic cancers, high cost, treatment resistance, and adverse side effects such as nausea, hair loss, immune suppression, and organ damage, due to their nonspecific targeting of both cancer and healthy cells [2,3,4,5,6]. Due to the limitations of current treatments, the search for new therapeutic strategies has become a primary focus in cancer research [7]. Natural compounds derived from plants, microorganisms, and marine organisms, because of their biological properties and relatively low toxicity profiles, are attractive candidates for drug development [8,9]. 

Phytochemicals, the bioactive compounds from plants, including alkaloids, flavonoids, polyphenols, and terpenoids, exhibit a wide range of antineoplastic activities, making them invaluable in cancer research [10,11,12,13,14,15]. By targeting multiple signaling pathways simultaneously, these compounds disrupt critical processes of cancer cells such as proliferation, invasion, angiogenesis, migration, and metastasis, and show immense chemosensitizing potentials [16,17,18,19,20,21,22]. Among the plethora of bioactive natural compounds, liquiritigenin (LIQ) has garnered significant attention in recent years, due to its potential anti-inflammatory, anti-oxidant, antidiabetic, and anticancer properties [23,24,25,26,27]. LIQ is a flavonoid primarily found in the roots of *Glycyrrhiza* species, commonly known as licorice [28,29]. This herb and its constituents have been part of various traditional herbal medicines worldwide, due to their immense bioactive properties [30,31]. 

Accumulating evidence suggests that LIQ exerts potent anticancer activities across various malignancies, including brain, breast, lung, oral, and prostate cancers, etc. [32,33,34,35,36,37]. Given its efficacy against a wide spectrum of diseases, LIQ has emerged as a promising candidate for further exploration in cancer treatment. Therefore, this review provides a comprehensive overview of the anticancer properties of LIQ, shedding light on its mechanisms of action in various cancer types. The chemistry and different natural sources of LIQ are discussed, providing the foundation for its pharmacological importance and therapeutic potential. Moreover, this review also focuses on the biological properties and mechanism of action of LIQ, which collectively contribute to its value as a future cancer drug. Through these comprehensive examinations, this review envisions the potential of LIQ in shaping effective cancer therapies.

## 2. Sources of LIQ

LIQ is an important phytochemical in various plant species, particularly those found in traditional medicines and dietary supplements. Licorice is the most well-known source of LIQ. *Glycyrrhiza* species, including *G. glabra*, *G. uralensis*, and *G. inflata*, belonging to the Leguminosae family, contain significant amounts of LIQ [28,30]. The term ‘*Glycyrrhiza*’ is derived from glykos, which means “sweet” and rhiza, which means “root” in Greek [30]. In addition, some notable sources of LIQ include *Dalbergia odorifera*, sprouts of alfalfa (*Medicago sativa*), etc., which have been summarized in Table 1.

## 3. Structure and Chemistry of LIQ

LIQ is a flavonoid, specifically a type of flavanone, which is a class of compounds known for their anti-oxidant properties [58,59]. Its chemical formula is C_15_H_12_O_4_, with a molecular weight of 256.25 g/mol. Structurally, LIQ is a 4′,7-dihydroxyflavanone with two hydroxy substituents at the 4′ and 7′ positions (PubChem ID 114829). LIQ is structurally and biochemically related to a chalcone, isoliquiritigenin, an isomeric precursor of LIQ [60]. Interestingly, Simmler et al. demonstrated that the interconversion of ILG and LIQ can be achieved in cell culture-based conditions, suggesting their interlinked biological activities [60].

## 4. Biological Properties and Mechanism of Action of LIQ

**Figure 1:**
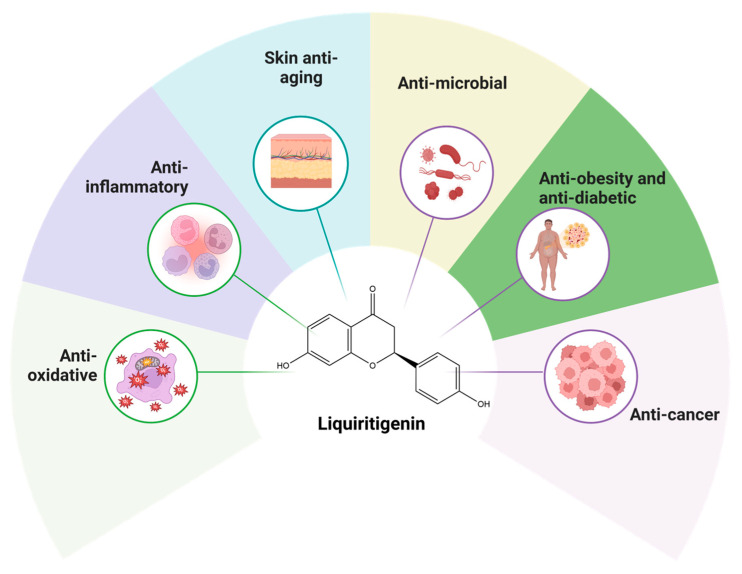
This figure illustrates the diverse biological properties of LIQ, highlighting its anti-inflammatory, anti-oxidative, anti-aging, antimicrobial, anti-obesity, antidiabetic, and anticancer properties. The figure also includes the molecular structure of LIQ, displayed at the center (PubChem ID 114829).

LIQ exhibits a wide array of biological activities that influence different health conditions and diseases (Figure 1).

Notably, LIQ is an excellent anti-oxidative agent, which plays a crucial role in counteracting free radicals that accumulate and cause oxidative stress in the body [61,62]. It is well known that oxidative stress is a major contributor to the development of several diseases, and LIQ, through its significant antioxidant activity, provides protection against conditions such as cardiovascular diseases, neuronal diseases, and cancer [35,61,63]. Further, several studies have shown that LIQ effectively mitigated various inflammation-induced disease states such as arthritis, kidney diseases, hepatotoxicity, and so on [23,26,29]. Additionally, Kim et al. demonstrated that LIQ exhibited significant anti-inflammatory effects by inhibiting nuclear factor kappa-light-chain-enhancer of activated B cells (NF-κB) activation in macrophages and the subsequent suppression of inducible NOS and pro-inflammatory cytokines, such as tumor necrosis factor alpha (TNF-α), interleukin (IL)-1β, and IL-6 [64]. Further, a couple of studies revealed that LIQ suppressed collagen-induced arthritis in rat and mouse models by inhibiting inflammation and associated cytokines [23,26].

Numerous studies have demonstrated that LIQ can modulate the inflammatory response associated with various chronic conditions, including non-alcoholic fatty liver disease (NAFLD), arthritis, asthma, hepatic sinusoidal obstruction syndrome (HSOS), etc. For example, Bao et al. revealed that LIQ treatment in high-fat diet-induced NAFLD mice resulted in improved lipid metabolism, reduced insulin resistance, and decreased inflammatory markers, potentially mediated through the activation of PI3K/Akt signaling pathway [65]. Another study reported that collagen-induced arthritis (CIA) mice, when treated with LIQ, displayed histopathological alterations in the synovium and serum [26]. This improvement was associated with reduced levels of pro-inflammatory cytokines, such as TNF-α, IL-1β, IL-6, and IL-17A. Additionally, LIQ treatment suppressed the expression of matrix metallopeptidases (MMP)-3 and MMP-13 in the synovium and reduced the level of fibrotic markers such as fibronectin, collagen I, and collagen III, in the cardiac tissues. Further, LIQ inhibited the expression of transforming growth factor-β1 (TGF-β1) and phosphorylated Smad2/3 in cardiac tissues, indicating a potential mechanism by which LIQ alleviates myocardial complications in CIA. These findings suggest that LIQ could be a promising therapeutic candidate for rheumatoid arthritis and its associated cardiac complications [26]. Similarly, another study demonstrated that LIQ attenuated inflammation in macrophages and collagen-induced arthritis in vivo [23]. Further, LIQ effectively suppressed the expression of various cytokines, modulated the NF-κB signaling pathway, influenced T-cell polarization, and elevated the levels of cyclic AMP (cAMP) in vitro, suggesting LIQ has the potential as an anti-asthmatic agent [66]. Furthermore, it was demonstrated that both LIQ and liquiritin ameliorated monocrotaline-induced HSOS in rats by attenuating hepatic inflammatory responses mediated by the activation of Nrf2 defense system [67].

LIQ has also shown protective effects against bone-related diseases, such as preventing bone loss, inhibiting osteoclast differentiation, and reducing adverse bone effects. For instance, LIQ promoted bone growth in ovariectomized (OVX) mice by enhancing osteogenic differentiation and modulating autophagy and apoptosis pathways, suggesting it could serve as a promising natural therapeutic option for osteoporosis [68]. Similarly, LIQ reduced osteoporotic phenotype in glucocorticoid-induced adult zebrafish by preventing osteoclast activation in scales [69]. In another study, it was observed that LIQ effectively inhibited osteoclast differentiation and bone-resorption activity in murine osteoblastic cells by moderately reducing the phosphorylation of extracellular signal-regulated kinase (ERK), c-Jun N-terminal kinase (JNK), and inhibitor of nuclear factor kappa Bα (IκBα) [70]. Conversely, the phosphorylation levels of Akt and p38 were slightly elevated in bone marrow-derived osteoclasts. Additionally, the expression levels of osteoclast marker proteins such as cathepsin K, nuclear factor of activated T-cell cytoplasmic-1 (NFATc1), and Src were reduced. These findings indicate that LIQ may serve as a plausible therapeutic agent for osteoporosis and other inflammatory bone diseases [70].

LIQ has been shown to activate various caspases in cancer cells, thereby promoting both intrinsic and extrinsic pathways of apoptosis in these cells [35,37,71]. Caspase-2 and -8 are the initiator caspases that trigger the downstream executioner caspase activation [72]. Liu et al. demonstrated that LIQ induced mitochondrial apoptotic pathway in cervical cancer cells, including the release of cytochrome c and the subsequent activation of effector caspases [73]. Moreover, LIQ’s close analog, isoliquiritigenin, has been reported to trigger caspase-8 in Ca Ski cervical cancer cells, followed by cytochrome c release, activation of the caspase cascade, and PARP cleavage [74]. Moreover, another study demonstrated that isoliquiritigenin induced p62/SQSTM1 upregulation and the subsequent activation of caspase-8-dependent apoptotic signaling pathway in HT-29 and SW-480 colon cancer cells [75]. While direct activation of caspase-2 by LIQ is yet to be elucidated, these initiator caspases, caspase-2 and caspase-8, trigger the execution phase of apoptosis, confirming that LIQ promotes programmed cell death through classical apoptotic mechanisms. In addition, Zhai et al. explored the impact of LIQ on bladder cancer, highlighting not only its inhibitory effects on tumor progression, but also its influence on intestinal microbiota and metabolomics. It was observed that the administration of LIQ in the murine model resulted in significant alterations to the gut microbiota, characterized by the upregulation of numerous beneficial bacterial genera, suggesting a multifaceted approach to cancer treatment involving gut health modulation [27]. Additionally, several studies have reported the anti-diabetic and anti-obese effects of LIQ [25,76]. Moreover, another study demonstrated the anti-skin aging potential of LIQ by promoting collagen synthesis and proliferation of keratinocytes [77].

Importantly, a few studies have reported that LIQ exerts epigenetic regulatory effects by modulating histone acetylation, deacetylation, and DNA methylation, which play critical roles in gene expression and tumor progression. For example, Liu et al. investigated the post-translational modifications (PTMs), of histones via a super stable isotope labeling by amino acids in cell culture (super-SILAC) strategy. LIQ enhanced histone acetylation at multiple lysine residues (e.g., H4K5ac, H3K9ac, H3K27ac) and simultaneously downregulated various histone deacetylases (HDAC4, HDAC5, HDAC8, SIRT2, SIRT3, SIRT7) in M1 macrophages [78]. Moreover, isoliquiritigenin was shown to enhance HDAC activity in HT-29 cells under TNF-α challenge and suppress the release of high-mobility group box 1 (HMGB1), a critical mediator of inflammation [79]. Additionally, Liang et al. demonstrated that LIQ reduced breast tumorigenicity by elevating DNA-damage-inducible 45 alpha (GADD45A) and suppressing DNA methyltransferase (DNMT) activity [80]. Further, Hua et al. demonstrated that LIQ activated the epigenetic regulator, sirtunin 1 (SIRT1), a well-known HDAC, in primary mouse myofibroblasts, thereby exerting anti-pulmonary fibrosis effects [81]. Furthermore, another study showed that LIQ enhanced the expression of SIRT3 in cisplatin-induced mouse models. This study highlighted the nephroprotective activity of LIQ and its potential as a therapeutic agent against acute kidney injury [82]. These studies highlight the epigenetic regulatory effects of LIQ, including DNMTs and HDAC enzymes, thereby emphasizing its therapeutic role in inflammation and cancer. 

In addition, several studies investigated the anti-bacterial and anti-fungal properties of LIQ. For instance, Gaur et al. demonstrated the ability of LIQ to reverse drug resistance in methicillin-resistant *Staphylococcus aureus*, providing a potential strategy to combat antibiotic-resistant bacterial infections. This indicates LIQ’s potential not only as a direct antibacterial agent, but also as an adjunct therapy to enhance the efficacy of existing antibiotics [83]. Moreover, another study demonstrated LIQ’s efficacy in enhancing immune response against disseminated candidiasis in mice, where this compound reduced colony-forming units in kidneys. This study suggested that LIQ could be used in anti-fungal formulations [84]. These studies collectively highlight LIQ’s potential as a multi-faceted agent capable of targeting various molecular mechanisms involved in different diseases, making it a candidate of interest for further therapeutic research and development.

Although flavonoids exhibit potential anti-inflammatory, anti-oxidant, antidiabetic, anticancer, and antimicrobial properties, a major limitation affecting their pharmacological application is their poor water solubility, which leads to limited oral bioavailability and rapid metabolism [85,86]. According to the biopharmaceutic classification system, low bioavailability of phytochemicals is primarily attributed to their limited solubility in water and low permeability through the cell membrane [87,88]. This notion paves the way for the development of artificial carriers for these flavonoids, to improve their penetration to target organs without affecting healthy tissues and organs [85]. The major nanosystems employed for the delivery of plant flavonoids include phytosomes, lipid-based nanoparticles, polymeric nanoparticles, and inorganic nanoparticles [86]. Notably, few studies have reported the enhanced bioavailability of LIQ when encapsulated in nanocarriers. For example, LIQ-loaded submicron emulsions exhibited a 595% increase in area under the curve (AUC) and significantly higher plasma concentration of 2831.17 ng/mL compared to free LIQ (210.84 ng/mL) in rats, demonstrating enhanced bioavailability and anti-oxidant activity [89]. In addition, a liquiritigenin–phospholipid complex (LPC) was developed, which, when administered to rats, showed 239% more plasma concentration–time curve (AUC0–t) than LIQ [90]. 

Apart from LIQ, its chalcone isoliquiritigenin has also shown enhanced bioavailability when encapsulated in nanocarriers. For instance, Zhang et al. demonstrated that the oral administration of isoliquiritigenin, loaded onto a nanostructured lipid carrier, to rats exhibited enhanced bioavailability of this compound. The plausible reasons for this may be the direct uptake of nanoparticles by the gastrointestinal tract, increased permeability by surfactants, and reduced intestinal clearance [91]. In addition, another study reported that isoliquiritigenin nanosuspensions showed improved cancer cell cytotoxicity and cellular uptake compared to isoliquiritigenin solution [92]. Moreover, these nanosuspensions resulted in decreased cell viability and 7.5–10-fold enhanced apoptotic rates in lung cancer cells compared to isoliquiritigenin. Furthermore, both the nanosuspensions and pure drug showed low cytotoxicity in normal human embryonic lung fibroblast (HELF) cells, which emphasizes their stronger activity in cancer cells with minimal effects in normal cells [92]. Further, another study revealed that licorice flavonoids nanoparticles (LFNs), including LIQ, showed higher bioavailability and dissolution rate than raw licorice flavonoids [93]. These studies highlight the promise of nanotechnology-based formulations to improve the therapeutic efficacy of LIQ.

## 5. Multifaceted Anticancer Effects of LIQ Across Diverse Cancer Types

**Figure 2:**
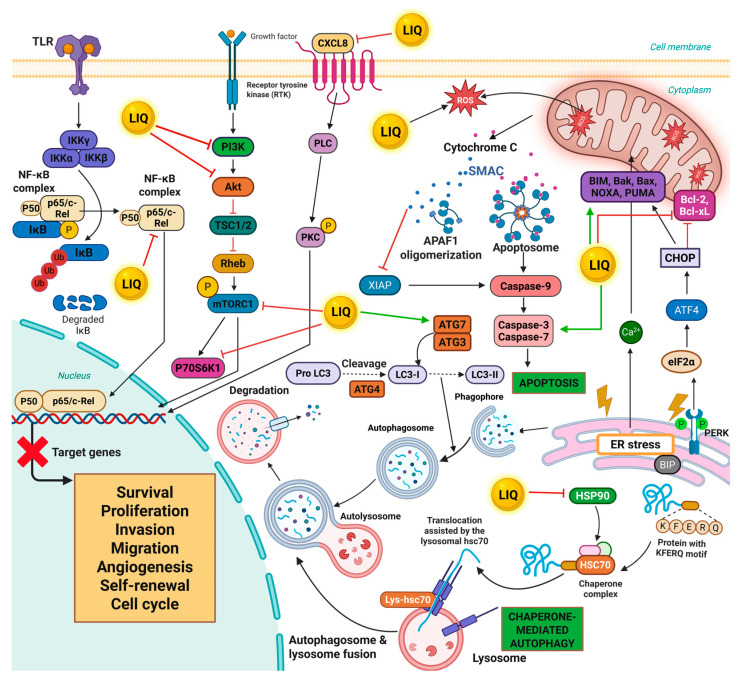
A schematic representation depicting the intricate molecular mechanism triggered within cancer cells following treatment with LIQ. LIQ exhibits its anticancer effects by suppressing key oncogenic signaling pathways, including major signaling pathways such as NF-κB, PI3K/Akt/ mTOR, and MAPK. Further, LIQ induces cell death by promoting apoptosis, characterized by caspase activation and mitochondrial dysfunction. It also triggers autophagy, including chaperone-mediated autophagy (CMA), a selective form of autophagy responsible for degrading specific cytosolic proteins to maintain cellular homeostasis. Collectively, these molecular alterations highlight the potential of LIQ as a promising therapeutic agent in cancer treatment.

LIQ has demonstrated versatile anticancer properties across a spectrum of malignancies. Its anticancer activities are mediated through multiple mechanisms, including induction of apoptosis, suppression of proliferation, and modulation of several signaling pathways which are crucial for the survival and metastasis of cancer cells (Figure 2).

**Figure 3:**
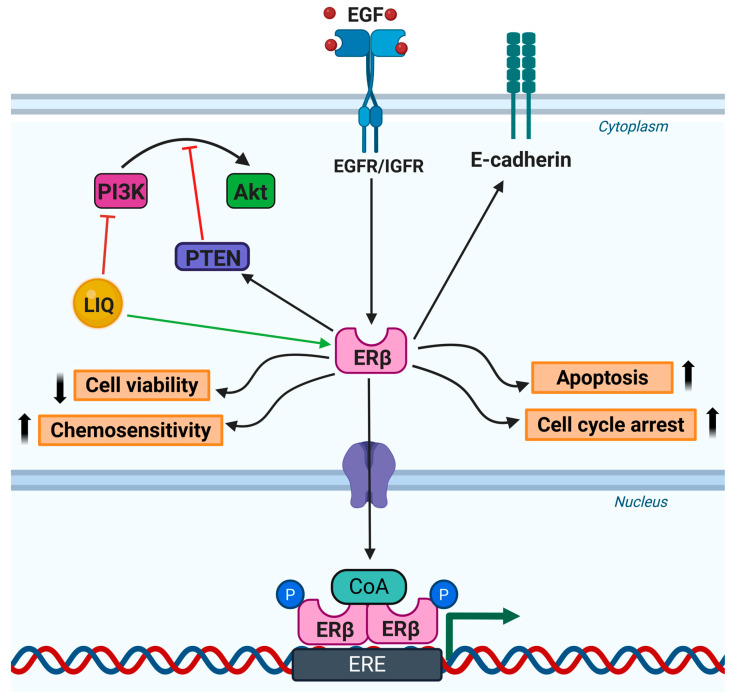
This figure illustrates the role of LIQ as a phytoestrogen in modulating cancer cell behavior by activating estrogen receptor β (ERβ). Upon binding to ERβ, LIQ suppresses cancer cell viability while promoting cell cycle arrest, apoptosis, and chemosensitivity of these cells. In addition, ERβ activation leads to the upregulation of phosphatase and tensin homolog (PTEN), a critical tumor suppressor. Simultaneously, LIQ downregulates the phosphoinositide 3-kinase (PI3K)/Akt signaling pathway, which is commonly associated with cell survival, proliferation, and drug resistance in cancer cells. Collectively, these molecular events contribute to the anticancer effects of LIQ (↑—Increase/Activation; ↓—Decrease/Suppression).

This section explores the anticancer activities of LIQ, highlighting its impact on different cancer cell types through diverse mechanistic pathways (summarized in Table 2). Further, LIQ has a phytoestrogenic role through which it activates ERβ, thereby modulating several cellular processes (Figure 3).

### 5.1. Breast Cancer

Breast cancer remains the most prevalent cancer among women worldwide, posing a significant health challenge [114]. According to 2022 GLOBOCAN statistics, breast cancer accounts for about 2.3 million new cases, representing 24.5% of all cancers diagnosed in women [1]. This cancer resulted in 670,000 deaths globally, in the year 2022 alone. In this context, the anticancer properties of LIQ could be a promising candidate for achieving these targets by enhancing therapeutic outcomes and reducing mortality rates among breast cancer patients. For instance, Xu et al. demonstrated that LIQ isolated from the plant *Polygonatum sibiricum* inhibited the proliferation, invasion, and migration of breast cancer cells. In addition, LIQ suppressed molecular chaperone heat shock protein 90 (HSP90), a pro-carcinogenic protein, which promoted breast cancer development through chaperone-mediated autophagy (CMA). Further, LIQ treatment reduced the levels of heat shock cognate 71 kDa protein (HSC70) and lysosome-associated membrane protein type 2A (LAMP-2A) in breast cancer cells and inhibited HSP90-mediated CMA [94]. Another study explored the CYP19A1 aromatase inhibitory potential of 14 flavonoids, including LIQ, for the treatment of hormone-dependent breast cancer, which was traditionally managed with aromatase inhibitors that often carry undesirable side effects. LIQ, along with other flavonoids such as chrysin, eriodictyol, naringenin, pinocembrin, and sakuranetin, demonstrated IC50 values under 10 μM. These compounds were further evaluated using in silico tools for their drug-likeness properties, indicating their strong potential for the development of flavonoid-based aromatase inhibitors for the therapeutic management of breast cancer [95]. In addition, another study reported that LIQ exhibited potential aromatase inhibitory effects in breast tissues of high-risk postmenopausal women, which is corroborated by molecular docking studies showing its effective binding in pockets of aromatase enzyme. Moreover, LIQ also resulted in reduced proliferation of breast cancer cells, thereby indicating its potential as a therapeutic agent for breast cancer in high-risk postmenopausal women [96]. In addition, Zhang et al. demonstrated that LIQ significantly reduced the expression of connective tissue growth factor (CTGF) and the growth, invasion, and migration of breast cancer cells in a dose-dependent manner. A dual-luciferase assay confirmed that miR-383-5p directly targets CTGF. It was found that LIQ inhibited proliferation, invasion, and migration of breast cancer cells by upregulating miR-383-5p and downregulating CTGF [97]. Another study revealed that LIQ and RO 48-8071, a small-molecule inhibitor of oxidosqualene cyclase, which is a cholesterol biosynthesis enzyme, independently showed a significant reduction in the viability of breast cancer cells. However, a combination of these resulted in a higher decrease in cell viability [98]. Further, their combinatorial treatment substantially suppressed ERα, but enhanced ERβ expression, and inhibited tumor growth in nude mice xenografts. Conclusively, the anti-tumor efficacy of RO 48-8071 may be partially attributed to an off-target modulation, where it diminishes the expression of ERα and augments that of ERβ. The upregulated ERβ interacts with LIQ, resulting in tumor-suppressive effects. Hence, this study indicated that the combination of RO 48-8071 and LIQ could be promising for developing innovative therapeutics for hormone-dependent breast cancer [98]. In addition, another study demonstrated that LIQ and 7-methoxy-LIQ inhibited the proliferation of breast cancer cells in a dose-dependent manner [99]. Another noteworthy study revealed that triple-negative breast cancer (TNBC) cells exhibited rapid invasive capabilities when co-cultured with osteoblast-like cells, and treatment with LIQ attenuated this invasion, to a great extent. LIQ treatment also suppressed the expression of CXC motif chemokine receptor 4 (CXCR4) protein, which plays a critical role in the invasion of tumor cells [100]. However, LIQ did not show any significant effect on the proliferation of TNBC cells. Mechanistically, LIQ functions as an ERβ-selective agonist, and impedes the ability of TNBC cells to invade the basement membrane and migrate. Hence, LIQ can be a potential therapeutic option to prevent bone metastasis in advanced breast cancer [100]. Moreover, Liang et al. reported that LIQ reduced the viability, invasion, migration and induced apoptosis in breast cancer cells. It also elevated the expression of BRCA1 and suppressed cellular DNMT activity, thereby exerting anti-breast cancer activities [80]. In addition, Lei et al. evaluated the effects of doxorubicin (DOX) and LIQ alone and in combination, in TNBC cells. LIQ treatment reduced the viability, colony formation, invasion, and migration potential of TNBC cells. Moreover, low concentrations of LIQ enhanced the sensitivity of these cells toward DOX and inhibited the PI3K/Akt/mTOR pathway in an ERβ-dependent manner. This study suggested that combining conventional therapeutics with natural ERβ agonists may effectively overcome chemoresistance in TNBC [32]. Further, another study demonstrated that LIQ treatment reduced the invasive potential of breast cancer cells [102]. Furthermore, another contrasting study investigated the selective estrogen receptor modulator (SERM) activity of a few dietary compounds, including apigenin, coumestrol, daidzein, genistein, LIQ, resveratrol, and zearalenone on ER-positive breast cancer cells. LIQ-treated cells showed increased estrogenic effects, resulting in enhanced proliferation of breast cancer cells and elevated expression of CXCL12 gene, a chemokine involved in cell proliferation [101]. Similarly, Lattrich et al. demonstrated that low concentrations of LIQ did not affect the proliferation of breast cancer cells. However, higher concentrations led to increased cell proliferation and enhanced the expression of cyclin B1, suggesting non-specific activation of ERα by LIQ [103].

The aforementioned studies indicate that LIQ exhibits dual functionality in breast cancer, wherein it sometimes suppresses tumor growth by acting as an ERβ agonist, yet, paradoxically, it promotes cell proliferation, probably due to unspecific activation of ERα and its estrogenic activity. Therefore, specific targeting is necessary to harness LIQ as a therapeutic agent, by overcoming its proliferative risks in breast cancer therapy.

### 5.2. Brain Cancer

Brain cancer, encompassing a wide spectrum of malignancies, is a significant clinical challenge due to its location and complexity [115]. Glioma stands out as a prevalent type of brain tumor that originates from the glial cells, with glioblastoma being the most aggressive and common subtype [116,117]. The year 2022 witnessed around 321,476 new cases of brain and central nervous-system tumors and 248,305 deaths, globally [1]. Despite advancements in multimodal treatments, which include radiation, surgery, and chemotherapy, survival of brain cancer patients has shown very little improvement [118]. Hence, there is a critical need for the development of innovative and more effective treatment regimes. LIQ has garnered attention for its potential therapeutic properties in the treatment of brain tumors, particularly gliomas and glioblastomas. For instance, Sareddy et al. evaluated the impact of ERβ signaling on glioma cells. This study found that LIQ enhanced ERβ expression, which significantly diminished stem-like properties of glioma stem cells (GSCs) [33]. Further, LIQ treatment inhibited neurosphere formation, cell proliferation, and self-renewal ability of GSCs. This compound also induced apoptosis and reduced the expression of stemness markers in GSCs. Furthermore, LIQ significantly reduced tumor growth in orthotopic mouse models and improved the overall survival of the mice [33]. In addition, another study investigated the role of ERβ agonists in gliomas. Glioma cells treated with LIQ decreased the proliferation of these cells via elevated expression of ERβ. Moreover, LIQ substantially reduced tumor size and volume in mice xenograft models, indicating the tumor-suppressive activities of ERβ [107]. Together, these studies highlight the importance of ERβ in glioma biology, and suggest that ERβ agonists could serve as a viable therapeutic approach to target glioma stem cells and possibly reduce tumor recurrence and improve survival. Notably, another study revealed that LIQ treatment could overcome temozolomide (TMZ) resistance in glioma cells. LIQ enhanced the expression of ERβ and sensitized glioma cells towards TMZ-induced inhibition of proliferation by modulating PI3K/Akt/mTOR signaling [106]. These studies highlighted the promising therapeutic roles of LIQ against different brain tumor types.

### 5.3. Colorectal Cancer

Colorectal cancer (CRC) ranks as the third most diagnosed cancer and second most lethal cancer worldwide, characterized by the uncontrolled growth of glandular cells within the colon or rectum [119]. Numerous studies have revealed that the major risk factors for CRC include genetic predisposition, lifestyle factors such as diet and smoking, and chronic inflammation [120,121,122,123]. Given the high prevalence of CRC and poor 5-year survival rate, novel treatment strategies are still needed to dovetail for the efficient management of this disease [17,119,124]. Notably, LIQ has been shown to reduce the malignant properties of CRC, making the exploration of its mechanisms of action important to promote its application in therapies for CRC. For instance, LIQ showed potential anti-proliferative effects on human colorectal adenocarcinoma cells, thereby emphasizing its therapeutic potential against CRC [105]. While the therapeutic potential of LIQ against CRC is promising, it is still in the early stages. Further research involving animal models and clinical trials is necessary to fully understand its efficacy and safety in humans and to determine appropriate dosages and potential combinations with other treatments, which could pave the way for innovative therapies that incorporate LIQ as a key component in the fight against CRC.

### 5.4. Liver Cancer

Liver or hepatic cancer is a deadly form of cancer, with its predominant form being hepatocellular carcinoma (HCC) [125]. According to GLOBOCAN 2022, liver cancer ranks sixth in terms of incidence and third in terms of mortality around the world, making it one of the most lethal cancer types [1]. Liver cancer prognosis remains poor, mainly due to its late-stage diagnosis. Despite conventional treatment strategies such as ablation, resection, and transplantation, there is a pressing need for more effective therapeutic options [126]. In this context, emerging evidence indicates the therapeutic potential of LIQ against liver cancer. For instance, Zhou et al. reported that LIQ inhibited tumor growth in hepatocarcinoma mouse models. This compound increased thymus weight in the mice, but not the spleen, and reduced MDA at moderate levels. Notably, LIQ imparted morphological changes in the nuclei of the tumor cells of the treated groups and showed signs of apoptosis [108]. Another study revealed that LIQ inhibited cell proliferation and induced apoptosis in HCC cells [37]. Caspase-3 activity and cleaved PARP expression were enhanced greatly, and the expression of B-cell lymphoma-2 (Bcl-2) and B-cell lymphoma-extra-large (Bcl-xL) was suppressed by LIQ. In addition, LIQ increased lactic acid dehydrogenase (LDH) release and promoted the phosphorylation of JNK and p38. In addition, LIQ inhibited ERK activation. Moreover, these antitumor activities of LIQ were further confirmed in mice xenograft models, where this flavonoid reduced tumor size. Conclusively, this study highlighted the anticancer potential of LIQ by activation of the MAPK signaling pathway [37]. These studies position LIQ as a promising candidate for further research and development in liver cancer therapy.

### 5.5. Lung Cancer

Lung cancer is the most commonly diagnosed cancer in the world, with around 2.5 million incidences and around 1.8 million deaths in the year 2022 [1]. It has two major subtypes—small-cell lung cancer (SCLC) and non-small-cell lung cancer (NSCLC) [127]. The majority of lung cancer cases are attributable to tobacco smoking [1]. Despite advancements in targeted therapies and immunotherapies, the survival rate of lung cancer patients is low, due to therapy resistance and late-stage diagnosis [128,129]. Therefore, more efficient treatment modalities have to be devised to combat this highly aggressive form of cancer. Multiple studies have shown that LIQ is effective in mitigating various hallmarks of lung cancer. For instance, Wang et al. demonstrated that LIQ inhibited adhesion and migration of lung adenocarcinoma cells [34]. It reduced the expression of promatrix metallopeptidase-2 (proMMP-2) and impeded phosphorylation of Akt. In addition, LIQ resulted in the phosphorylation and activation of ERK1/2 [34]. Moreover, another study reported that LIQ inhibited the viability, proliferation, and colony-forming ability of lung squamous-cell carcinoma cells in a dose-dependent manner [110]. Additionally, LIQ induced apoptosis, disruption of mitochondrial membrane potential, and cell cycle arrest in these cells. Further, LIQ inhibited tumor growth in mice models. Furthermore, LIQ suppressed levels of PI3K, Akt, and mTOR in lung cancer cells, suggesting that this compound induced anti-tumor activities by the inhibition of PI3K/Akt/mTOR signaling pathways [110]. Moreover, Khamsan et al. also reported that LIQ shows inhibitory effects on the viability of SCLC cells [53]. Collectively, these studies demonstrated that LIQ could be a plausible agent for the treatment of lung cancer.

### 5.6. Ovarian Cancer

Ovarian cancer is a complex and often lethal gynecological malignancy, resulting in an increased number of incidences and deaths in women worldwide [1]. It is characterized by late-stage diagnosis and high molecular heterogeneity, making it one of the deadliest female reproductive cancers [1,130]. Moreover, adverse effects of treatment strategies such as chemotherapy have also affected the quality of life of patients, to a great extent [130,131]. Notably, LIQ has shown substantial protective effects in ovarian cancer. In addition, LIQ’s potential to synergize with existing chemotherapeutics has also been explored. For instance, Liu et al. investigated the therapeutic potential of natural ERβ selective agonists, LIQ and S-equol, in ovarian cancer [71]. LIQ significantly inhibited viability, and induced apoptosis, in ovarian cancer cells. These compounds also decreased the invasion and migration potential of these cells. RNA sequencing and gene set enrichment analysis (GSEA) revealed that LIQ suppressed the expression of genes that are correlated with NF-κB signaling. In addition, this natural ERβ agonist downregulated the NF-κB pathway and its target genes, IL-1β, C-X-C motif chemokine ligand 8 (CXCL8), and prostaglandin-endoperoxide synthase 2 (PTGS2), in vitro. Notably, LIQ sensitized therapy-resistant ovarian cancer cells to paclitaxel and cisplatin treatment [71]. Moreover, LIQ reduced tumor growth in xenograft models, supporting its potential as a therapeutic agent in ovarian cancer management. Conclusively, this study indicated that LIQ could be an effective therapeutic agent, targeting signaling pathways involved in ovarian cancer progression and overcoming chemotherapy resistance [71]. Further, another study also revealed the inhibitory effects of LIQ on the proliferation of ovarian cancer cells [112]. These antitumor characteristics position LIQ as a candidate for further investigation in the context of integrative therapies aimed at improving outcomes in ovarian cancer treatment.

### 5.7. Prostate Cancer

Prostate cancer is one of the most common types of cancer among men, ranking fourth in terms of incidence and eighth in terms of mortality, worldwide [1]. It originates in the prostate gland, which produces seminal fluid [132]. Age, familial history, factors related to diet, and hormone profiles are among the major risks for prostate cancer [133]. LIQ has shown potential therapeutic effects against prostate cancer. For instance, a recent study explored the anticancer properties of LIQ in prostate cancer cells [36]. This study revealed that LIQ inhibited invasion, EMT, and migration, in these cells, which are critical processes in metastasis, in a dose-dependent manner. Treatment with LIQ led to elevated levels of the epithelial marker, E-cadherin, and decreased mesenchymal markers, N-cadherin, and vimentin. This study indicated that LIQ could be a plausible therapeutic option for the treatment of prostate cancer, particularly for targeting its metastatic behavior [36]. However, more in vitro, in vivo, and clinical studies are required, to explore the potential of LIQ for its integration into existing prostate cancer regimens.

### 5.8. Other Cancers

In addition to the cancers discussed previously, LIQ has also demonstrated potential tumor-suppressing effects in several other types of cancer, including cervical, oral, melanoma, pituitary, and laryngeal cancers. For instance, LIQ treatment reduced tumor weight and volume in nude mice xenograft models of cervical cancer. Further, LIQ decreased the microvascular density of the tumor and the expression of vascular endothelial growth factor (VEGF) [104]. In addition, Frozza et al. demonstrated that treatment of laryngeal cancer cells with red propolis fraction containing LIQ led to the appearance of apoptotic bodies, and resulted in DNA fragmentation and chromatin condensation in these cells. Hence, this study indicated the efficacy of LIQ fractions against laryngeal cancer [109]. Further, Wang et al. revealed that LIQ elevated intracellular ROS and cytosol cytochrome C levels, inhibited cell proliferation, and induced cell cycle arrest and apoptosis in pituitary adenoma cells. Additionally, this phytochemical attenuated the expression of Bcl-2, Bcl-xL, and Ras, and inhibited the activation of ERK in these cells. Moreover, LIQ inhibited tumor growth in mice xenograft models [113]. Another study reported that the treatment of LIQ resulted in significant anticancer effects, such as suppression of cell proliferation and promotion of apoptosis and autophagy in oral cancer cells. Additionally, it targets and inactivates the PI3K/Akt/mTOR signaling pathway, a critical regulator of cell growth and survival [35]. Moreover, in vivo studies replicate these findings, showing that LIQ inhibits tumor growth and induces apoptosis and autophagy in animal models, suggesting LIQ’s potential as a therapeutic agent in oral cancer [35]. In addition, Shi et al. investigated the effect of the combinatorial treatment of LIQ and cisplatin on melanoma cells and its mechanistic underpinnings. The findings of this study revealed that LIQ sensitized melanoma cells towards cisplatin-induced cytotoxicity. Further, this combination reduced the viability, invasion, and migration of melanoma cells and suppressed lung metastasis in mouse models [111]. Hence, these studies collectively denote the potential of LIQ as an efficient therapeutic agent against various cancers.

## 6. Pharmacokinetics of LIQ

LIQ has been renowned for its therapeutic properties, including anti-inflammatory, anti-oxidant, antidiabetic, and anti-allergic activities, making it a promising candidate for the management of various diseases [25,67,134,135]. However, the poor bioavailability of LIQ, due to its low solubility in water, limits its efficacy in in vivo models [89,90]. In addition, it has also been proved that increased sulfonation at the 7-hydroxyl group of LIQ might account for its poor bioavailability [136]. Kang et al. investigated the factors contributing to the low oral bioavailability of LIQ and its major glucuronide conjugates, M1 (4′-O-glucuronide) and M2 (7′-O-glucuronide), in rats. The results revealed that, following a 20 mg/kg oral dose, LIQ exhibited a bioavailability of only 6.68%, primarily due to extensive gastrointestinal first-pass metabolism, which was responsible for approximately 92.5% of the oral dose being lost before reaching systemic circulation [137]. 

Following oral administration in rats, LIQ metabolized into five conjugates: 4′-O-glucuronide, 7-O-glucuronide, 4′,7-O-disulfate, 4′-O-glucuronide-7-O-sulfate, and 7-O-glucuronide-4′-O-sulfate [138]. Subsequently, it was found that liver and kidneys are the major organs involved in the metabolism of LIQ, along with a rapid disappearance from plasma and bile, confirming the immediate uptake of LIQ, fast conjugation, and rapid efflux of conjugates out of the organs [138]. Notably, in rats, intravenous administration of 50 mg/kg LIQ resulted in a rapid decline in the plasma. Additionally, LIQ also exhibited hepatoprotective activity characterized by the activation of phase II enzymes and hepatic transporters [139]. Another study examined the metabolic fate of LIQ by incubating it with liver and gut microbiota isolated from rats, using concentrations of 1 µmol/mL and 20 μg/mL, respectively [140]. The liver microbiota converted LIQ into two primary metabolites: 7,4′-dihydroxyflavone and naringenin, while the gut microbiota produced phloretic acid, resorcinol, and a compound identified as M5. These findings further confirm that extensive metabolism, particularly by hepatic and intestinal microbiota, likely contributes to the poor oral bioavailability of LIQ [140]. Another study investigated the role of stereospecificity in the metabolism and elimination of LIQ in rat models [141]. S-LIQ showed shorter half-life in serum when compared to R-LIQ, and the elimination of LIQ was through non-renal routes. It has also been found that LIQ undergoes rapid glucuronidation upon intravenous administration [141]. A similar study reported that the intravenous administration of LIQ containing both racemic mixtures led to a high volume of distribution for R-LIQ, as compared to S-LIQ, in rat tissues [142]. 

Another study evaluated the intestinal absorption of LIQ and other compounds, including davidigenin, liquiritin, and liquiritin apioside, using Caco-2 cells. LIQ and davidigenin showed high permeability and excellent absorption [143]. Another study examined the pharmacokinetic profiles of four flavanones derived from *Glycyrrhiza*, including LIQ. Rats were administered 0.468 g/kg of the flavanone mixture, and blood samples were collected at designated intervals for analysis, using HPLC. Results showed that LIQ reached a peak plasma concentration of 2.83 ± 0.02 μg/mL and demonstrated rapid absorption and distribution, along with the slowest elimination rate among the flavanones tested [144]. Moreover, Kang et al. reported that administration of LIQ to animals, such as mice, rats, rabbits, and dogs, showed a positive correlation of total body clearance and volume distribution with body weight [145].

In addition, Kang et al. administered LIQ and its conjugates, M1 and M2, to diabetic rat models via intravenous (20 mg/kg) and oral (50 mg/kg) routes. In diabetic rats, intravenous administration resulted in a higher plasma AUC (26.6 ± 21.6 μg·min/mL) compared to control rats (17.8 ± 9.12 μg·min/mL), likely due to altered intestinal metabolism associated with the diabetic condition [146]. In a subsequent study, using hepatitis rat models under similar conditions, intravenous administration of LIQ resulted in a lower plasma AUC compared to controls, which was attributed to enhanced hepatic clearance or increased metabolism of LIQ into its M2 conjugate [147]. Furthermore, in a renal-failure rat model, LIQ exhibited reduced urinary excretion, a 19.7% increase in terminal half-life, and an 87.0% decrease in clearance rate compared to control rats, indicating impaired renal elimination [148]. Similarly, in a fibrosis mouse model, treatment with a multi-component composition of huangqi decoction containing LIQ (2.016 mg/kg) showed hepatoprotective activity by reducing serum aminotransferase activity and hepatic collagen fibril deposition [149]. Moreover, the plasm concentration of LIQ was less than 1% of glycyrrhetinic acid present in the decoction, and the elimination half-life was less than 3 h [149].

## 7. Discussion

This review explores the multifaceted roles of the flavonoid LIQ in the realm of cancer research and therapy. LIQ and its natural sources have been shown to have a rich historical background in traditional and herbal medicine. In addition, a plethora of pre-clinical research has shown that LIQ exhibits a broad spectrum of biological activities, including anti-oxidant, anti-inflammatory, anti-apoptotic, anti-aging, antimicrobial, hepatoprotective, and other effects. Importantly, this phytochemical has potential estrogenic activity, where it selectively recruits co-activators of ERβ and acts as a selective ERβ agonist [150]. Notably, LIQ exhibits a 20-fold higher affinity for ERβ than ERα, highlighting its potential as a targeted therapeutic agent in hormone-related cancers [150]. 

LIQ’s anticancer activity is underpinned by its diverse mechanisms across different cancer types. For instance, it has been shown to inhibit cell proliferation and induce apoptosis by activating the various signaling pathways. Further, it acts as an inhibitor of cell cycle in cancer cells by modulating the expression of cyclin-dependent kinases and cyclins, thereby disrupting the normal cell cycle. Furthermore, LIQ exerts its antitumor potential by inhibiting several signaling pathways, such as NF-κB, PI3K/Akt/mTOR, MAPK, JNK, and their associated proteins, which play crucial roles in cancer progression. LIQ substantially inhibited the expression levels of different proteins involved in several processes of cancer progression, such as proliferation, invasion, angiogenesis, migration, EMT, etc.

It has to be noted that the selectivity of LIQ towards cancer cells can be attributed to its capacity to modulate abnormally regulated signaling pathways, which are hallmarks of malignancy. Many aforementioned studies indicate that LIQ exerts selective cytotoxic effects on cancer cells by downregulating pro-survival pathways such as PI3K/Akt and NF-κB, which are frequently overexpressed in cancer. Moreover, Liu et al. demonstrated, through RNA-Seq analysis, that the NF-κB signaling pathway is prominently modulated in response to LIQ treatment. Further, NF-κB and its target genes were observed to be significantly downregulated in ES2, SKOV3, and SKOV3 (taxol-resistant) cells [71]. Furthermore, Liu et al. demonstrated that LIQ selectively exerted its anticancer effects in lung squamous-cell carcinoma cells, such as SK-MES-1 and NCI-H520, with no significant cytotoxicity on BEAS-2B normal-lung epithelial cells [110]. On the contrary, Yang et al. reported that LIQ attenuated early apoptosis in HT22 neuronal cells, thereby exhibiting neuroprotective effects [151]. Moreover, another study revealed that treatment of RAW264.7 mouse macrophage cells with different concentrations of LIQ did not affect the viability of these cells up to 100 μM [64]. These findings emphasize that LIQ’s anticancer effects are selective to cancer cells, dependent on the aberrant molecular context of these cells, and exhibit minimal toxicity or protective effects on healthy tissues. Additionally, LIQ enhances the sensitivity of cancer cells to chemotherapeutic drugs by modulating drug-resistance pathways. This chemosensitizing effect makes LIQ a plausible agent to be used as an adjuvant in conventional chemotherapy regimens, thereby improving treatment outcomes.

The evaluation of a therapeutic agent’s safety and efficacy is critical to determining its suitability for clinical development. In the case of LIQ, both in vitro and in vivo studies provide encouraging evidence regarding its favorable safety profile, alongside its potent anticancer efficacy. Several studies have reported that LIQ exhibits low toxicity at therapeutically relevant doses. For instance, Zhang et al. revealed that LIQ at concentrations ranging from 0.05 to 0.8 mmol/L inhibited the proliferation of breast cancer cells without affecting normal mammary epithelial cells, MCF-10A [97]. Such differential sensitivity is an essential attribute for anticancer agents, as it minimizes adverse effects and enhances therapeutic precision. Further evidence supporting the safety of LIQ comes from toxicological studies, where LIQ showed no systemic toxicity in animal models. For example, Li et al. investigated the effects of LIQ in a bladder-cancer mouse xenograft model, and found that LIQ significantly inhibited tumor growth without inducing any observable toxicity. Comprehensive histopathological evaluations of vital organs, including the liver, heart, lungs, kidneys, and spleen, demonstrated no structural abnormalities, thereby reinforcing the compound’s biocompatibility and non-toxic nature in vivo [152]. Moreover, Wang et al. investigated the toxicity of LFNs, including LIQ, in Sprague Dawley rats, for 14 days. The rats were administered with 100 mg/kg, 400 mg/kg, and 800 mg/kg of LFNs by gavage, and different organs such as heart, liver, spleen, lungs, and kidneys were examined. Histopathological analysis revealed that LFNs did not show toxicity in the rats up to 800 mg/kg [93]. These findings are especially important, as they suggest that LIQ selectively targets malignant tissues without causing collateral damage to normal physiological systems.

Various preclinical investigations mentioned in this review provide insights into the concentration range at which LIQ exhibited its potential therapeutic benefits. For instance, Xu et al. reported that LIQ inhibited breast cancer cell growth at 0.2 mmol/L [94]. Further, Liang et al. demonstrated that LIQ treatment significantly decreased the viability of breast cancer cells at 20 μM [80]. Another study demonstrated that LIQ, when treated in a concentration range of 0–200 μM, showed potential inhibitory effects on melanoma cell growth at 100–200 μM range [111]. Moreover, Liu et al. revealed that 80 μM of LIQ enhanced the efficacy of temozolomide (TMZ), sensitizing glioma cells to the chemotherapeutic agent [106]. Similarly, Wang et al. reported a significant reduction in hepatoma cell growth at concentrations ranging from 200 to 500 μM. In vivo studies further corroborated these findings, where administration of LIQ at a dose of 20 mg/kg effectively suppressed tumor progression in nude mice xenograft models [37]. Further, Ji et al. demonstrated that LIQ inhibited tumor growth in oral cancer xenograft models at 20 mg/kg [35]. Together, these studies suggest that LIQ exhibits anticancer efficacy across a broad concentration range, typically from 100 μM to 400 μM in vitro and around 20 mg/kg in vivo, depending on the cancer type. The findings from these dose-dependent preclinical studies provide a critical foundation for the development of LIQ as a safe and efficacious anticancer compound. However, clinical studies are warranted for the successful translation of LIQ into a therapeutic agent for cancer therapy.

## 8. Conclusions

LIQ is a compound of significant interest, due to its diverse biological activities and potential therapeutic applications in cancer treatment. Its mechanisms of action are comprehensive, targeting multiple pathways crucial in cancer progression and chemotherapy resistance. Future research, particularly clinical trials, is essential to validate these preclinical findings and to explore the full potential of LIQ for its integration into cancer therapy strategies. By bridging the gap between traditional remedies and modern medicine, LIQ presents a promising avenue for novel anticancer therapies that could offer enhanced efficacy and reduced toxicity for patients suffering from various cancers.

## 9. Future Perspectives

Despite LIQ’s potential anticancer activities, several challenges have to be addressed before its clinical translation. Also, few clinical trials have been conducted to analyze the pharmacokinetic properties of various formulations that contain LIQ as a major component. For instance, in 30 healthy volunteers, oral administration of huangqi decoction containing LIQ exhibited an elimination half-life of less than 3 h, which is in line with the results from mice models [149]. In a similar study, urine samples were collected from healthy volunteers orally administered with Saiboku-To, a Chinese herbal formulation, to understand the components present in it [153]. However, large variations were found in the quantity of components present in urine among individuals, with a bimodal excretion profile for LIQ, at 1 h and 6–12 h [153]. Further, rikkunshito, a traditional Japanese medicine for the treatment of gastrointestinal disorders, has also been tested in 21 healthy volunteers in a three-arm cross-over study, to understand the pharmacokinetics of the ingredients [154]. Subjects were divided into three groups containing seven volunteers in each group, and orally administered a daily dose of 2.5 g, 5 g, and 7.5 g of rikkunshito. LIQ present in this formulation exhibited plasma AUC as 1180 pg.h/mL, 2560 pg.h/mL, and 4040 pg.h/mL, and median Tmax as 4 h, 3 h, and 3.02 h for 2.5 g, 5 g, and 7.5 g, respectively [154]. These results were in accordance with the results from in vivo models. Nevertheless, the low solubility of LIQ in water and lipids hinders its bioavailability. Hence, strategies involving nanoparticle-based delivery systems should be further explored to improve its systemic retention, leading to enhanced therapeutic effects of this compound. In addition, co-treatment with other natural bio-enhancers could also be a potential strategy to overcome the poor bioavailability of LIQ.

However, the limited number of clinical studies restricts the research of LIQ in in vivo systems. Therefore, it is imperative to conduct comprehensive clinical trials to elucidate the efficacy of LIQ in human physiology and metabolism, facilitating its development as a therapeutic agent. Hence, more in vivo and clinical studies are critical to validate LIQ’s safety and long-term therapeutic effects. Although LIQ’s antitumor potential has been demonstrated in preclinical settings, well-designed phase I and phase II clinical trials are necessary to evaluate its therapeutic benefits, toxicity profile, and pharmacodynamic properties in cancer patients. In addition, the synergistic anticancer effects of LIQ when combined with conventional chemotherapy and immunotherapy strategies should also be focused on, wherein this phytochemical overcomes the cancer drug-associated toxicities. Moreover, its role in modulating the tumor microenvironment should also be explored in detail. Although LIQ holds great promise as an anticancer agent, a multi-disciplinary approach should be adopted to fully harness its therapeutic potential and expedite its integration into clinical cancer research.

## Figures and Tables

**Table 1:** Different sources of LIQ.

Name of the Plant	Part Used	Amount of LG	References
*Glycyrrhiza uralensis*	Roots	13.8 mg	[38]
*Dalbergia odorifera*	Heartwood	2.70 mg/g	[39]
*Medicago sativa*	Sprouts	2.1 mg	[40]
*Maackia amurensis*	Heartwood	-	[41]
*Boerhavia erecta*	Aerial parts	3.7 mg	[42]
Brazilian red propolis	-	30 mg	[43]
Brazilian red propolis extracts	-	-	[44]
*Dalbergia ecastaphyllum*	Leaves	2.012 ± 0.025%/100 g	[44]
*Helianthus tuberosus*	Aerial parts	-	[45]
*Astragalus bhotanensis*	Roots	7.2 mg	[46]
*Rhus verniciflua*	Bark	15 mg	[47]
*Pterocarpus marsupium*	Heartwood	-	[48]
*Angelica keiskei*	Aerial parts	-	[49]
*Bauhinia ungulata*	Roots, stem	-	[50]
*Artocarpus heterophyllous*	Wood	20.8 mg	[51]
*Verbascum blattaria*	Leaves	-	[52]
*Jacaranda obtusifolia*	Twigs	2.2 mg	[53]
*Cotinus coggygria*	Heartwood	2 mg	[54]
*Ocimum basilicum*	Seeds	-	[55]
*Piptadeniastrum africanum*	Stem bark	-	[56]
*Astragalus mongholicus*	Flowers	0.34 μg/g	[57]

**Table 2:** Anticancer effects of LIQ alone or in combination with other compounds.

Intervention	In Vitro/In Vivo	Model	Mechanisms/Outcomes	References
Breast cancer				
LIQ	In vitro	MCF-7, BT20 cells	↑ E-cadherin ↓ Cell viability, colony formation, invasion, migration, Snail, HSP90, LAMP-2A, HSC70, Chaperone-mediated autophagy	[94]
LIQ	-	Human CYP19A1 supersomes	↓ Aromatase (CYP19A1)	[95]
LIQ	In vitro	Breast tissue microstructures of high-risk menopausal women	↓ Aromatase (CYP19A1)	[96]
LIQ	In vitro	MCF-7 cells	↓ Cell proliferation	[96]
LIQ	In vitro	BT483, AU565, BT20 cells	↑ Apoptosis, miR-383-5p ↓ Cell viability, invasion, migration, CTGF	[97]
LIQ + RO	In vitro	BT474, MCF-7 cells	↓ Cell viability	[98]
LIQ + RO	In vivo	Athymic nude mice (BT474 cells) xenograft	↑ Tumor clearance, Apoptosis, ERβ ↓ Tumor volume, size, ERα, VEGF, CD31	[98]
7-methoxy-LIQ	In vitro	MCF-7 cells	↓ Cell proliferation	[99]
LIQ	In vitro	HCC1806, HCC1937 cells (co-cultured with MG63 osteoblast-like cells)	↓ Cell invasion, CXCR4	[100]
LIQ	In vitro	MDA-MB-231, BT549 cells	↑ Apoptosis, Caspase-3, E-cadherin, BRCA1, p21, GADD45A, %cells in G1 phase ↓ Cell viability, colony formation, N-cadherin, vimentin, MMP-9, invasion, migration, EMT, DNMT1, DNMT3a, DNMT3b	[80]
LIQ + DOX	In vitro	MDA-MB-231, BT549 cells	↑ Sensitivity to DOX, ERβ ↓ Cell viability,	[32]
LIQ	In vitro	MDA-MB-231 cells	↓ Number of colonies, PI3K/Akt/ mTOR signaling, *p*-Akt/Akt ratio, *p*-mTOR/mTOR ratio	[32]
LIQ	In vitro	MCF-7, T47D (ER-positive) cells	↑ Cell number, CXCL12	[101]
LIQ	In vitro	MDA-MB-231 cells	↓ Invasion	[102]
LIQ	In vitro	MCF-7 cells	↑ Cell number, Cyclin B1, PS2	[103]
Cervical cancer				
LIQ	In vivo	BALB/c nude mice xenograft (HeLa cells)	↓ Tumor weight, volume, VEGF, MVD, PCNA-positive cells	[104]
Colorectal cancer				
LIQ	In vitro	HT-29 cells	↓ Cell survival	[105]
Brain cancer				
LIQ	In vitro	GSC10, GSC11 cells (Glioblastoma stem cells)	↑ Apoptosis ↓ Cell viability, neurosphere formation, self-renewal ability, nestin, SOX2	[33]
LIQ	In vivo	Athymic nude mice xenograft (U251-GSCs)	↑ Mice survival ↓ Tumor growth	[33]
LIQ + TMZ	In vitro	U138 cells	↑ Sensitivity to TMZ, ERβ ↓ Cell viability, *p*-Akt, *p*-P70SK6	[106]
LIQ	In vitro	U87, LN229, T98G, U138 cells	↓ Cell proliferation	[107]
LIQ	In vitro	U87, LN229	↑ G2/M phase arrest ↑ ERβ ↓ Number of colonies	[107]
LIQ	In vivo	Nude mice xenograft (U87 cells)	↑ Apoptosis, ERβ ↓ Tumor growth, PCNA	[107]
Oral cancer				
LIQ	In vitro	SCC-9, CAL-27 cells	↑ Apoptosis, cleaved Caspase-3&-9, autophagy, LC3II, ATG7, Beclin 1 ↓ Cell proliferation, Ki-67, PCNA, PI3K p85α, *p*-Akt, *p*-mTOR	[35]
LIQ	In vivo	BALB/c nude mice xenograft (CAL-27 cells)	↑ Apoptosis, autophagy, Beclin 1^+^ cells ↓ Tumor growth, weight, volume, *p*-Akt, Ki-67^+^ cells	[35]
Liver cancer				
LIQ	In vivo	ICR mice allograft (Ascites H_22_ cells)	↑ Body weight, thymus weight, necrosis ↓ Tumor volume	[108]
LIQ	In vitro	HepG2, PLC/PRF/5 cells	↑ Intracellular LDH, Apoptosis, Caspase-3, cleaved PARP, JNK, p38, ROS ↓ *p*-ERK, Bcl-2, Bcl-xL	[37]
LIQ	In vivo	BALB/c athymic nude mice xenograft (PLC/PRF/5 cells)	↓ Tumor size	[37]
Laryngeal cancer				
Red propolis fractions containing LIQ	In vitro	Hep2 cells	↑ Apoptotic bodies, DNA fragmentation, chromatin condensation	[109]
Lung cancer				
LIQ	In vitro	A549 cells	↑ *p*-ERK1/2 ↓ Cell adhesion, migration, proMMP-2, *p*-Akt	[34]
LIQ	In vitro	SK-MES-1, NCI-H520 cells	↑ G2/M phase cells, p21, p27, Apoptosis, Bak, Bax, Cleaved caspase-3, cleaved PARP ↓ Cell viability, proliferation, Ki-67, Bcl-2, Bcl-xL, Mcl-1, PCNA, Cyclin B1, CDK1, *p*-PI3K, *p*-Akt, *p*-mTOR	[110]
	In vivo	BALB/c nude mice xenograft (SK-MES-1 cells)	↓ Tumor growth	[110]
LIQ	In vitro	NCI-H187 cells	↓ Cell viability	[53]
Melanoma				
LIQ	In vitro	B16F10 cells	↓ Cell viability	[111]
LIQ + CDDP	In vitro	B16F10 cells	↑PTEN ↓ Cell viability, invasion, migration, MMP-2&-9, PI3K, *p*-Akt,	[111]
LIQ	In vivo	C57BL/6 mice allograft (B16F10 cells)	↑ PTEN ↓ Invasion, migration, *p*-Akt, PI3K metastatic nodules, MMP-2&-9	[111]
Ovarian cancer				
LIQ	In vitro	SKOV3, ES-2 (cisplatin-resistant), BG-1, SKOV3 (taxol-resistant) cells	↓ Cell viability	[71]
LIQ	In vitro	SKOV3, ES-2 cells	↓ Cell viability, invasion, migration, colony formation	[71]
LIQ	In vitro	SKOV3, ES-2 (cisplatin-resistant), SKOV3 (taxol-resistant) cells	↑ Caspase-3/-7	[71]
LIQ + Paclitaxel, LIQ + Cisplatin	In vitro	ES-2, SKOV3 cells	↑ Sensitivity to paclitaxel and cisplatin	[71]
LIQ	In vitro	ES-2, SKOV3 cells	↓ NF-κB, IL-1β, CXCL8, PTGS2	[71]
LIQ	In vivo	Nude mice xenograft (SKOV3 cells)	↑ Apoptosis ↓ Tumor weight, volume, tumor nodules, Ki-67, IL-1β, COX-2	[71]
LIQ	In vitro	OAW-42 cells	↓ Cell viability, ND6	[112]
LIQ	In vitro	OVCAR-3 cells	↑ GAS2 ↓ Cell viability, CCNE2	[112]
Pituitary adenocarcinoma				
LIQ	In vitro	MMQ, GH3 cells	↑ Apoptosis, G1 phase arrest, ROS ↓ Cell viability, Bcl-2, Bcl-xL, Ras, *p*-ERK	[113]
LIQ	In vivo	BALB/c athymic nude mice xenograft (GH3 cells)	↓ Tumor size	[113]
Prostate cancer				
LIQ	In vitro	C4-2, PC3 cells	↑ E-cadherin, ER stress, IRE1, ATF6, BIP ↓ Cell proliferation, invasion, migration, N-cadherin, vimentin	[36]
LIQ + TUDCA (Stress inhibitor)	In vitro	C4-2, PC3 cells	↑ E-cadherin, ER stress, IRE1, ATF6, BIP ↓ Invasion, migration, N-cadherin, vimentin	[36]
LIQ + shIRE1	In vitro	C4-2, PC3 cells	↑ Invasion, migration, N-cadherin, vimentin ↓ E-cadherin, IRE1	[36]
LIQ	In vivo	Nude mice xenograft (PC3 cells)	↑ E-cadherin, IRE1, BIP ↓ Tumor weight, volume, N-cadherin, lung metastasis	[36]

↑—Increase/Activation; ↓—Decrease/Suppression.

## Data Availability

Not applicable.

## Abbreviations

AKR—Aldoketo reductases; AUC—Area under the curve; Bcl-2—B cell lymphoma-2; Bcl-Xl—B-cell lymphoma-extra-large; cAMP—Cyclic adenosine monophosphate; CDDP—Cis-diamine dichloroplatinum; CDK1—Cyclin-dependent kinase 1; COX-2—Cyclooxygenase-2; CRC—Colorectal cancer; CTGF—Connective tissue growth factor; CXCL8—C-X-C motif chemokine ligand 8; CXCR4—C-X-C motif chemokine receptor 4; DNMT1—DNA Methyltransferase 1; DOX—Doxorubicin; EMT—Epithelial–mesenchymal transition; ER—Estrogen receptor; ERK—Extracellular signaling-regulated kinases; GADD45A—Growth arrest and DNA-damage-inducible 45 alpha; GAS2—Growth arrest specific 2; GFAP—Glial fibrillary acidic protein; GRM3—Glutamate metabotropic receptor 3; GSEA—Gene set enrichment analysis; HSOS—Hepatic sinusoidal obstruction syndrome IARC—International Agency for Research and Cancer; IL—Interleukin; JNK-c—Jun N-terminal kinases; LFN—Licorice flavonoid nanoparticles; LDH—Lactate dehydrogenase; LIQ—Liquiritigenin; Mcl-1—Myeloid cell leukemia sequence 1; MVD—Microvascular density; NF-κB—Nuclear factor kappa-light-chain-enhancer of activated B cells; PARP—Poly (ADP-ribose) polymerase; PCNA—Proliferating cell nuclear antigen; PI3K/Akt/mTOR—Phosphatidylinositol-4,5-bisphosphate 3-kinase/Akt/mammalian target of rapamycin; ProMMP—Promatrix metalloproteinase; PTEN—Phosphatase and TENsin homolog deleted on chromosome 10; PTGS2—Prostaglandin-endoperoxide synthase 2; ROS—Reactive oxygen species; Runx2—Runt-Related Transcription Factor 2; SOX2—Sex-determining region Y-box 2; VEGF—Vascular endothelial growth factor; TUDCA–Tauroursodeoxycholic acid
